# Adipokines and Their Role in Intestinal Inflammation

**DOI:** 10.3389/fimmu.2018.01974

**Published:** 2018-10-12

**Authors:** Carl Weidinger, Jörn F. Ziegler, Marilena Letizia, Franziska Schmidt, Britta Siegmund

**Affiliations:** ^1^Charité – Universitätsmedizin Berlin, Corporate Member of Freie Universität Berlin, Humboldt-Universität zu Berlin, Berlin Institute of Health, Medizinische Klinik für Gastroenterologie, Infektiologie und Rheumatologie, Berlin, Germany; ^2^Clinician Scientist Program, Berlin Institute of Health, Berlin, Germany

**Keywords:** adipokines, inflammatory bowel disease, Crohn's disease, creeping fat, immune modulation

## Abstract

Fat tissue was initially described for its endocrine and metabolic function. Over the last two decades increasing evidence indicated a close interaction with the immune system. Partly responsible for this immune modulatory function are soluble factors released by the fat tissue, most prominently the so-called adipokines. These discoveries led to the question how adipokines influence inflammatory diseases. Linking inflammation and adipose tissue, Crohn's disease, a chronic inflammatory bowel disease, is of particular interest for studying the immune modulatory properties of adipokines since it is characterized by a hyperplasia of the mesenteric fat that subsequently is creeping around the inflamed segments of the small intestine. Thus, the role of several adipokines in the creeping fat as well as in intestinal inflammation was recently explored. The present review selected the four adipokines adiponectin, apelin, chemerin, and leptin and provides a working model based on the available literature how these factors participate in the maintenance of intestinal immune homeostasis.

## Introduction

Adipokines represent a group of mediators primarily released by adipocytes that modulate a variety of metabolic functions within the fat tissue and additional organs such as liver, brain and muscle. In addition to the metabolic functions, a regulatory role within the immune system was identified early on for a number of adipokines. This includes adiponectin which in first reports was shown to suppress mature macrophage function (1) as well as leptin that was identified as a T cell stimulatory factor (2). These initial publications induced a plethora of studies exploring the impact of various adipokines in several inflammatory diseases. Why is intestinal inflammation of particular interest for the regulatory function of adipokines?

In Crohn's disease, one subtype of inflammatory bowel disease (IBD), characteristic changes of the mesenteric fat suggest that the mesenteric fat might play a central immune modulatory role in the pathogenesis of Crohn's diseases (3). In this disease, the mesenteric fat becomes hyperplastic and is creeping around the inflamed segments of the small intestine, suggesting a direct role of this fat tissue compartment in Crohn's disease. In order to provide a concise overview how adipokines regulate intestinal inflammation, we here selected adipokines for which experimental data in intestinal inflammation were available. Thus, we chose apelin, adiponectin, chemerin, and leptin. In order to include the relevant literature, we chose the names of the respective adipokine in combination with either colitis, IBD, Crohn's disease, or ulcerative colitis as search terms in PubMed. This review will first focus on the selected adipokines (summary provided in Table 1), namely apelin, adiponectin, chemerin and leptin, and then provide a disease-relevant working model.

**Table 1:** Adipokines and the regulatory effect on intestinal homeostasis.

**Adipokine**	**Effect on mucosal homeostasis**	**References**
Adiponectin	*Function* Adiponectin plays a key role in regulating insulin sensitivity. In addition, an anti-inflammatory effect has been demonstrated for atherosclerosis.	(4)
	*in vitro* Globular adiponectin has been shown to enhance intestinal epithelial cell proliferation and prevented these cells from apoptosis. In line, *adiponectin^−/−^* mice developed more severe DSS-induced colitis accompanied by a decreased epithelial proliferation, increased apoptosis and cellular stress. This could be reversed *in vitro* in the presence of adiponectin.	(5, 6)
	*in vivo models* The data on the *in vivo* role are controversial. Fayad et al. provided evidence suggesting a pro-inflammatory role for adiponectin in DSS- as well as TNBS-induced colitis. Conversely, Nishihara et al. found the opposite. In addition, work by Sideri et al. demonstrated that silencing of AdipoR1 was followed by deterioration of TNBS-induced colitis. These obvious discrepancies might be explained by differences in knockout mice.	(7–10)
	*Human data* Adiponectin has been shown to be upregulated in the creeping fat of Crohn's disease as compared to non-creeping fat of Crohn's disease, ulcerative colitis and healthy controls.	(11)
Apelin	*Function* Apelin induces proliferation of intestinal epithelial cells. Apelin plays a role in the development and stabilization of lymphatic vessels.	(12)
	*in vivo models* Apelin is upregulated in the intestinal epithelial cells of colitic mice. Apelin administration ameliorated colitis in *Il10^−/−^* mice by enhancing intestinal lymphatic function.	(13, 14)
	*Human data* Apelin is upregulated in the intestinal epithelial cells of IBD patients. Apelin is highly expressed in the mesenteric adipose tissue of Crohn's disease patients.	(12, 15)
Chemerin	*Function* Chemerin has been shown to serve as chemoattractant for cells of the innate immune system.	(16)
	*in vivo models* Administration of chemerin aggravated DSS-induced colitis and was associated with a decrease in anti-inflammatory macrophages. Accordingly, mice deficient in the chemerin receptor, chemokine-like receptor 1 (CMKLR1), develop colitis in a delayed course, ultimately resulting in similar disease severity.	(17, 18)
	*Human data* Chemerin was elevated in the serum of IBD patients.	(19)
Leptin	*Function* Leptin was initially identified as a hormone and satiety factor. Early work revealed an enhancing effect on T cell proliferation and polarization.	(2, 20)
	*in vitro* Leptin has been shown to induce an increased intestinal epithelial cell proliferation. In addition, intestinal epithelial cells produce leptin and luminal leptin *vice versa* activates the NF- κB pathway in intestinal epithelial cells. In line, rectal application *in vivo* results in intestinal inflammation. Leptin signaling is required for *in vitro* polarization of T_h17_ cells as leptin receptor (Lepr) deficient T cells display decreased STAT3 signaling and consecutively less RORγt expression and impaired IL-17 and IFNγ production.	(21–23)
	*in vivo models* Leptin-deficient *ob/ob* mice are protected from experimental colitis. Colitis induction depends on the activation of T cells by leptin as proven in the T cell transfer model of colitis. Leptin produced by T cells does not contribute to colitis development.	(24–27)
	*Human data* Leptin is upregulated in the mesenteric fat of Crohn's disease patients. The presence of leptin in the mesenteric fat favors the polarization of tissue macrophages toward an anti-inflammatory phenotype.	(28, 29)

*AdipoR1, adiponectin receptor type 1; DSS, dextran sodium sulfate; IBD, inflammatory bowel diseases; NF- κB, nuclear factor “kappa-light-chain-enhancer” of activated B-cells; TNBS, trinitrobenzene sulphonic acid*.

## Adipokines

### Apelin

Early on, apelin has been shown to be elevated in the colonic tissue of mice suffering from colitis as well as in humans with IBD. Apelin was mainly expressed by epithelial cells and addition of synthetic apelin in cell culture increased epithelial cell proliferation (12). More recently, apelin was described to play a significant role in the development as well as the stabilization of lymphatic vessels (13, 14). Remarkably, defects in the lymphatic transport of the mesentery have previously been suggested to be involved in the pathogenesis of Crohn's disease. This is reflected by an increased density of lymphatic vessels as well as the presence of tertiary lymphoid organs in the mesentery of Crohn's disease patients (30, 31). Accordingly, apelin was shown to be highly expressed in the mesenteric adipose tissue of Crohn's disease patients. In addition, the administration of apelin to *Il10*^−/−^ mice with established colitis resulted not only in an amelioration of disease by lowering the production of pro-inflammatory cytokines such as TNFα, IL-6, and IL-1β but furthermore enhanced intestinal lymphatic function. This was shown by an increased lymphatic vessel density. In addition, lymphangiography indicated an augmented lymphatic drainage (15). Based on these findings, one can propose a regenerative function of apelin on intestinal epithelial cells as well as a supportive role with regard to intestinal lymphatic drainage.

### Adiponectin

The adipokine adiponectin plays a key role in regulating insulin sensitivity and had previously been shown to play an anti-inflammatory role in atherosclerosis (4). The study by Yamamoto et al. analyzed the adiponectin expression in the creeping fat of Crohn's disease patients, that revealed an upregulation of adiponectin expression in creeping fat of Crohn's disease patients when compared to non-creeping fat of Crohn's disease patients as well as fat from ulcerative colitis patients or controls (11).

A study by Fayad et al. explored the function of adiponectin in intestinal inflammation by subjecting adiponectin-deficient animals to two models of experimental colitis, the model of dextran sulfate sodium (DSS)- and the model of trinitrobenzene sulphonic acid (TNBS)-induced colitis. Adiponectin was shown to rather induce the production of pro-inflammatory cytokines in the colon as adiponectin stimulation of colon organ cultures of DSS-treated mice resulted in increased production of IL-6 and MIP-2, whereas adiponectin-deficient APN knock out mice were protected from experimental colitis. Thus, these data were somewhat contrary to the findings described for atherosclerosis (8). A second study by Nishihara et al. explored again adiponectin-deficient mice in the DSS and TNBS model of colitis. In contrast to the previous study, adiponectin protected from inflammation, possibly mediated by a direct anti-inflammatory effect on the colonic epithelial cells (7). These discrepancies between the two studies as well as in several *in vitro* studies are most likely due to different types of knockout mice or adiponectin used (9).

In line with the first study, Ogunwobi and colleagues provide data analyzing the effect of adiponectin on colon epithelial cells by using the colonic epithelial cell line HT-29 and by carefully distinguishing globular adiponectin from full-length adiponectin. Remarkably, in particular globular adiponectin mediated pro-proliferative as well as pro-inflammatory effects through activation of extracellular-signal regulated kinase (ERK), p38 mitogen-activated protein kinase (MAPK) NF-κB signaling on colonic epithelial cells (5). When the human NCM60 epithelial cell line was exposed to fat-conditioned media obtained from IBD patients, cells showed a reduced expression of adiponectin receptor 1 (AdipoR1). Silencing of AdipoR1 in mice resulted in an exacerbation of TNBS-induced colitis (10).

In a recent study, adiponectin-deficient mice treated with DSS exhibited more severe colitis accompanied by an increased presence of activated B cells, pro-inflammatory cytokines such as IL-1β, IL-4, and IL-6 and increased STAT3 signaling in the colon. The epithelium of the knockout animals revealed a decrease in cell proliferation as well as increased apoptosis and cellular stress. In *in vitro* experiments these effects could be reversed by adiponectin. These data are supporting the concept that adiponectin maintains intestinal homeostasis (6).

### Chemerin

Chemerin has been shown to serve as chemo-attractant for cells of the innate immune system (16). Serum from Crohn's disease (*n* = 230), ulcerative colitis patients (*n* = 80), and healthy controls (*n* = 80) was recently compared for the expression of chemerin and adiponectin. Chemerin was elevated in IBD patients, whereas adiponectin was decreased (19). An *in vivo* study furthermore indicated that the administration of chemerin resulted in aggravation of DSS-induced colitis in mice by augmenting TNFα and IL-6 production, whereas a decrease in IL-10—producing anti-inflammatory macrophages could be detected. This could be confirmed *in vitro*, where the presence of chemerin prevented macrophages from polarizing into an anti-inflammatory phenotype resulting in impaired expression of Arginase-1 and IL-10 (17). The chemokine-like receptor 1 (CMKLR1) is the receptor for chemerin. Mice deficient in CMKLR1 developed DSS-induced colitis in a delayed time course, albeit ultimately presenting with a similar disease activity (18).

### Leptin

Leptin was initially identified as a satiety factor (20, 32). Work by Lord and colleagues revealed that the adipokine leptin supports T cell proliferation and results in increased T helper cell type 1 (T_h1_) and in suppressed T_h2_ cytokine production. In addition, administration of leptin to starved mice resulted in an abrogation of starvation-induced immunosuppression (2). The additional characterization of three children with congenital leptin deficiency indicated that leptin substitution not only reversed their metabolic dysfunction including insulin resistance and fatty liver degeneration, but furthermore increased the number of circulating CD4^+^ T cells as well as their proliferative capacity (33). These findings raised the question whether leptin exerts a modulating effect on autoimmune diseases. First evidence was provided from the model of experimental autoimmune encephalomyelitis. Here, leptin-deficient (*ob/ob*) mice were protected from T cell-mediated neuronal damage and leptin substitution augmented the disease susceptibility in mice. Additional work on the same model revealed that CNS-infiltrating T cells themselves are able to produce leptin and that this effect could be blocked by an anti-leptin receptor antibody (34, 35).

In an early study, systemic leptin concentrations were determined during the course of acute experimental colitis. In this study, the concentration of leptin in the plasma increased in TNBS-induced colitis and indomethacin-induced ileitis and correlated with disease severity. However, this observed increase was only transient and returned to control concentrations over time (36). The association of a distinct disease to plasma levels has proven difficult for several disease entities in the past. However, independent studies revealed a strong up-regulation of leptin expression in the mesenteric fat of Crohn's disease patients (28, 37).

In our own work, we were able to demonstrate that leptin-deficient *ob/ob* mice are protected from DSS-induced colitis and that leptin administration reverses disease susceptibility in mice (24). To confirm the previously established concept that leptin mediates pro-inflammatory effects, at least partly, via T cells, we performed a T cell transfer model of colitis and transferred naive CD4^+^ T cells lacking the signaling Ob-Rb-isoform of the leptin receptor. In fact, the development of colitis was significantly delayed using this approach, indicating that the stimulatory effect of leptin plays a crucial role in this model (25). Additional data showing that *ob/ob* mice are protected in models driven by either T_h1_ (TNBS) or T_h2_ (oxazolone) cells underline that the T cell-stimulating capacity of leptin is important for the observed effects (27). However, this enhancing factor does not apply for all T cell subpopulations, since leptin has been shown to inhibit the proliferation of regulatory T (T_reg_, FoxP3^+^CD4^+^CD25^+^) cells. Accordingly, the absence of leptin, as demonstrated for *ob/ob* and *db/db* mice (mice deficient for the signaling Ob-Rb isoform of the leptin receptor), resulted in an increased proliferation of functional T_reg_ cells (38). Likewise, Reis and colleagues could demonstrate that *Lepr*^*fl*/*fl*−^*CD4-Cre* mice harboring a conditional knock out of the leptin receptor in their CD4^+^ T cell compartment also showed higher frequencies of FOXP3^+^ T_reg_ cells under steady state conditions. Importantly, *Lepr*-deficient T cells displayed a severe defect in T_h17_ differentiation due to a decreased activation of the STAT3 signaling cascade, resulting in impaired cytokine production of IL-17 and IFNγ, which protected recipient *Rag-*^−/−^ mice receiving *Lepr*-deficient T cells from transfer colitis (23).

Data from the experimental autoimmune encephalomyelitis model have previously indicated that leptin produced by T cells might play a key role in mediating disease severity (23, 35). Thus, we performed again the T cell transfer model of colitis and chose this time naive T cells isolated from *ob/ob* mice as disease-inducing cell population in comparison to wild-type cells. In contrast to the data cited above, no differences were observed with regard to disease severity or cytokine production, implying that leptin produced by T cells is irrelevant for intestinal inflammation (26).

Recent data indicate that the administration of a pegylated leptin antagonist (PG-MLA) was sufficient to protect from chronic experimental colitis. Amelioration of colitis was here associated with a decrease in the expression of mucosal pro-inflammatory cytokines and an increase of mucosal T_reg_ cells (39).

Having shown that leptin modulates intestinal inflammation via the T cell compartment, the question arose whether there is an additional effect on epithelial cells. The leptin receptor (Ob-Rb) is in fact expressed in human colonic tissue as well as on the HT-29 colon cancer cell line. Stimulation of HT-29 cells with leptin was followed by activation of p42/44 MAPK as well as increased proliferation *in vitro* and *in vivo* (21). An interesting aspect was added by the study of Sitaraman and colleagues that were able to demonstrate that inflamed colonic epithelial cells produce and release leptin into the intestinal lumen. The presence of luminal leptin resulted in the activation of the NF- κB pathway in intestinal epithelial cells. Rectal application of leptin was followed by intestinal inflammation and epithelial wall damage (22). Following up on these luminal effects, Hoda et al. analyzed the effect of luminal leptin on ion transport capacities under inflammatory conditions. Human intestinal epithelial cells (T84) as well as intestinal tissue from a rat model of chemotherapy-induced enterocolitis were analyzed in Ussing chambers. These served to determine the transepithelial short-circuit current I(sc). The presence of leptin resulted in an increase of basal I(sc) of T84 cells, which was mediated by activation of the MAPK pathway. In line, in the enterocolits model luminal leptin equally induced an increase in I(sc), that was more prominent in the proximal colon (40).

A recent study was able to link leptin, cell metabolism and intestinal epithelial function. The authors provided evidence indicating that leptin is able to induce lipid droplet formation in intestinal epithelial cells (IEC-6). This was accompanied by an increased production of CXCL1/CINC-1, CCL2/MCP-1, and TGFβ. In line with previous data, leptin induced cell proliferation. The process of lipid droplet induction and the associated effects depended on the mammalian target of rapamycin (mTOR) pathway, since it was completely abrogated in the presence of rapamycin (41). Thus, one can conclude that leptin, very similar to adiponectin, exerts direct effects on intestinal epithelial cells and consequently plays a role in intestinal homeostasis.

## How gets the mesenteric fat tissue stimulated and adipokines released?

The question arises how the mesenteric fat gets involved in the first place. Courageous proposals suggest that changes in the mesenteric fat tissue might in fact be the initiating event of Crohn's disease. However, this is not only difficult to proof but it also seems to be more likely that the transmural inflammation and consecutive chronic bacterial translocation is the initiating factor for these changes. Crohn's disease, in contrast to ulcerative colitis, presents with a transmural inflammation and can also affect the small intestine. Several experimental models as well as studies in humans provided evidence of an increased translocation of bacteria into the mesenteric fat (42, 43). Previous data had already proven that adipocytes as well as preadipocytes express functional receptors of the innate immune system including toll-like receptors (Tlr) and nucleotide oligomerization domains 1 (NOD1) and 2 (NOD2) (44, 45). Accordingly, while wild type mice featured an up-regulation of pro-inflammatory mediators in chronic DSS colitis, *Myd88*^−/−^ mice, thus mice with a dysfunctional innate immune system, failed to respond to these translocalizing bacteria and showed a significantly increased mortality, suggesting that the mesenteric fat, serves as a potential second barrier (42, 46). In line, wild-type mice revealed an up-regulation of leptin in the mesenteric fat whereas this was not observed in *Tlr9*^−/−^ mice. However, IL-6 production was unaffected by the absence of Tlr9 (47), indicating that other innate receptors contribute to the effects described before (42).

What is the subsequent effect of adipokines produced within the mesenteric adipose tissue? Leptin has been shown to not only modulate the polarization of the T helper cell compartment but to equally affect together with adiponectin the myeloid compartment. Leptin as well as adiponectin favored the polarization of anti-inflammatory macrophages, the dominant macrophage population detectable in creeping fat of Crohn's disease patients (29). In addition, as described above for mice (42) and patients with Crohn's disease (43), the mesenteric fat develops an effector response to the translocalizing antigens. Remarkably, a dysfunction of this effector response, as discussed above for the *Myd88*^−/−^ mice, results in an increased mortality, thus suggesting a primarily protective function. Together this fits into the concept that the mesenteric fat represents a second barrier and thereby prevents the Crohn's disease patient from continuous bacteremia (48, 49).

**Figure 1:**
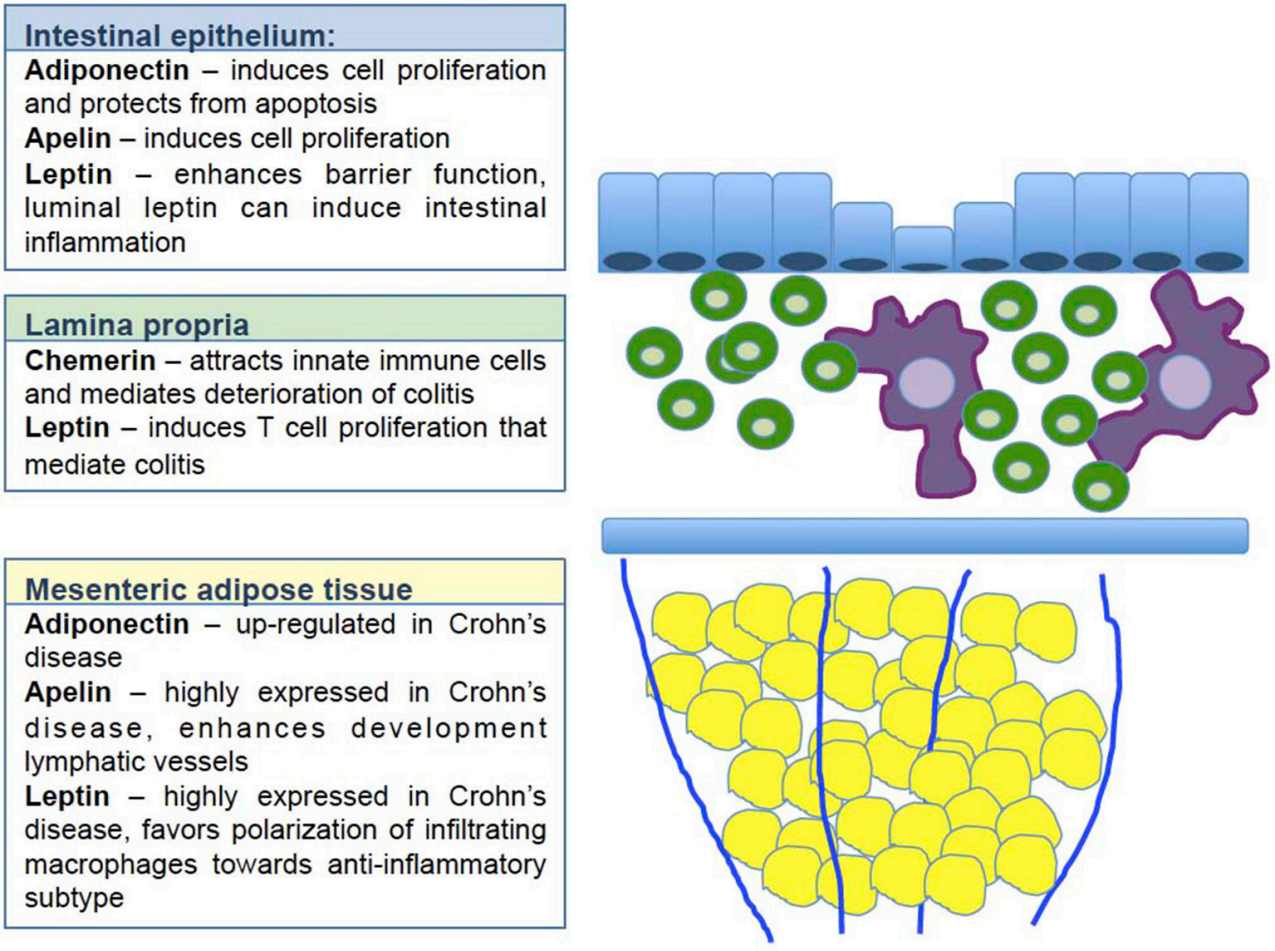
Regulatory effect of selected adipokines on intestinal homeostasis. The figure illustrates at which layer of the intestinal wall the selected adipokines exert their respective regulatory function. Blue indicates the intestinal epithelial layer. Adiponectin, apelin and leptin have been shown to enhance cell proliferation and enhance barrier function. The majority of these data derive from *in vitro* data, thus it remains open how these adipokines contribute to the complex crosstalk between epithelial cells and immune cells *in vivo*. Green indicates the lamina propria with all cell populations included. Chemerin has been shown here to attract innate immune cells resulting in a deterioration of colitis. Leptin induces T cell proliferation thus enhancing inflammation. Yellow indicates the mesenteric fat tissue. Adiponectin, apelin and leptin are all up-regulated in this compartment. Apelin enhances the function of the lymphatic vessels, that are known to be dysfunctional in Crohn's disease. Leptin strongly influences the polarization of infiltrating monocytes toward rather anti-inflammatory macrophages.

A summary of the effects mediated by adipokines is provided in Figure 1.

## Conclusions

Continuous work over the last two decades has substantially provided insights into the regulation of adipokines and intestinal inflammation. The data discussed for adiponectin, apelin, chemerin, and leptin within this review indicate a complex specific function for each individual adipokine in the regulation of intestinal inflammation. However, the summary also reveals the shortcomings of our current understanding. Thus, the complex interplay between the adipose tissue and the immune system presents a hot topic for current research. In our view, it is rather unlikely that adipokines will serve as therapeutic option for patients with IBD. Nevertheless, the overall metabolic condition of a patient, e.g., body mass index, presence of diabetes, might strongly influence the adipokine response on one hand, on the other hand, anti-inflammatory treatment in IBD patients might result in direct changes of the adipokine and thus metabolic profile. Therefore, this particular cross-regulation will become even more important in the foreseeable future.

## Authors contributions

JZ, ML, and FS contributed distinct parts of the manuscript. CW participated in writing and finalizing the manuscript. BS wrote the first draft and finalized the manuscript.

### Conflict of interest statement

BS served as consultant for Abbvie, Falk, Janssen, Hospira, MSD and Takeda and received speaker's fees from Abbvie, Falk, Ferring, Hospira, Janssen MSD, Takeda. BS received a research grant from Pfizer. All money went to the Charité.

The remaining authors declare that the research was conducted in the absence of any commercial or financial relationships that could be construed as a potential conflict of interest.
